# P-827. Effects of RSV Testing in a Community Hospital Setting: Quantitative and Narrative Analysis

**DOI:** 10.1093/ofid/ofaf695.1035

**Published:** 2026-01-11

**Authors:** Robert Colgrove, Shreya Arora

**Affiliations:** Mount Auburn Hospital, Harvard Medical School, Cambridge, Massachusetts; Mount Auburn Hospital, Harvard Medical School, Cambridge, Massachusetts

## Abstract

**Background:**

Prior to the COVID-19 pandemic, respiratory virus testing beyond influenza was uncommon for immunocompetent adults, with limited impact on clinical care. The pandemic ushered in much broader testing, including widespread deployment of rapid, in-house/point-of-care multiplex PCR platforms that simultaneously assay for SARS-CoV-2, Influenza A&B, and Respiratory Syncytial Virus (RSV). Understanding the clinical effects of such testing is of particular interest for RSV, since in prior years these infections would largely have gone undetected. This study evaluated how RSV detection, generated as a by-product of COVID testing, affected clinical decision-making in a medium-sized, community teaching hospital without RSV-specific protocols.

**Figure:**
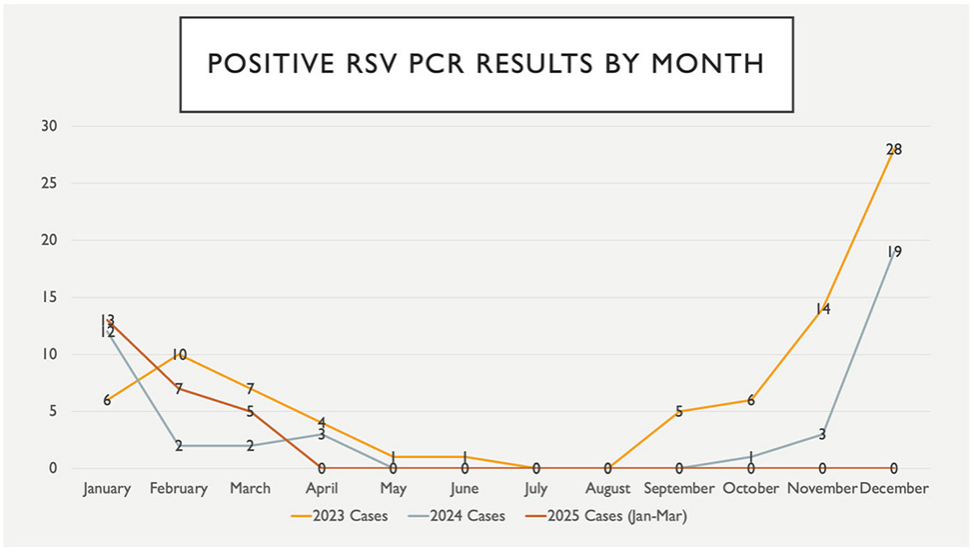

**Figure:**
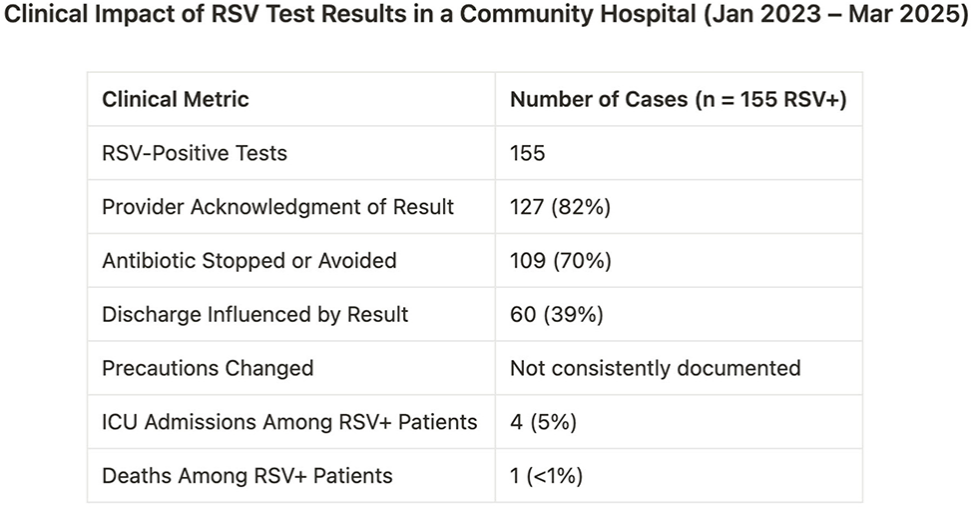

**Methods:**

We conducted a quantitative analysis and in-depth chart review of 1,694 positive cases from January 2023 to March 2025, tested using the Cepheid GeneXpert® multiplex PCR platform. Pathogen counts included RSV (n=155) [figure 1], influenza A (n=557), influenza B (n=39), SARS-CoV-2 (n=922), with 21 coinfections. We reviewed ICU admission, mortality, and clinical responses to RSV results: (1) provider acknowledgment, (2) antibiotic changes, (3) isolation adjustment, (4) impact on discharge.

**Results:**

RSV positivity frequently influenced care [table 1]: 82% of patients had provider acknowledgment of the result. For 70%, the positive RSV result was cited in stopping or avoiding antibiotics. Among Emergency Department-tested RSV patients, 39% were discharged directly post-result. ICU admission for RSV was 5%. Mortality was rare (n=1). Median test turnaround was 1.1 hours. Though isolation decisions were inconsistently charted, provider notes often referenced de-escalated imaging and consults following rapid RSV diagnosis.

**Conclusion:**

PCR detection of RSV, as part of a broader program of COVID screening, can influence clinical decisions in a substantial fraction of cases. Rapid, in-house testing of a previously less-tested pathogen such as RSV may yield substantial benefits by improving antimicrobial stewardship, discharge efficiency, and provider confidence. Even in the absence of RSV-specific therapy, diagnostic clarity proved clinically useful in a community hospital setting.

**Disclosures:**

All Authors: No reported disclosures

